# Validation of a mixture of rK26 and rK39 antigens from Iranian strain of *Leishmania infantum* to detect anti-*Leishmania* antibodies in human and reservoir hosts

**DOI:** 10.1038/s41598-022-14490-6

**Published:** 2022-06-21

**Authors:** Bibi Razieh Hosseini Farash, Mehdi Mohebali, Bahram Kazemi, Abdolmajid Fata, Homa Hajjaran, Behnaz Akhoundi, Reza Raoofian, Pietro Mastroeni, Elham Moghaddas, Azad Khaledi, Ghodratollah Salehi Sangani

**Affiliations:** 1 Department of Parasitology and Mycology, School of Medicine, Mashhad University of Medical Sciences, Mashhad, Iran; 2 Cutaneous Leishmaniasis Research Center, Mashhad University of Medical Sciences, Mashhad, Iran; 3 Department of Medical Parasitology and Mycology, School of Public Health, Tehran University of Medical Sciences, Tehran, Iran; 4 Center for Research of Endemic Parasites of Iran, Tehran University of Medical Sciences, Tehran, Iran; 5 Zoonosis Research Center, Tehran University of Medical Sciences, Tehran, Iran; 6 Faculty of Medicine, Shahid Beheshti University of Medical Sciences, Tehran, Iran; 7 Legal Medicine Research Center, Legal Medicine Organization, Tehran, Iran; 8 Department of Veterinary Medicine, University of Cambridge, Cambridge, United Kingdom; 9 Department of Microbiology and Immunology, School of Medicine, Kashan University of Medical Sciences, Kashan, Iran; 10 Faculty of Medicine, Mashhad University of Medical Sciences, Mashhad, Iran

**Keywords:** Microbiology, Diseases, Medical research

## Abstract

Mediterranean type of visceral leishmaniasis (VL) is a zoonotic parasitic infection. Some provinces of Iran are endemic for VL while other parts are considered as sporadic areas. This study aimed to assess a combination of recombinant K26 and rK39 antigens as well as crude antigen (CA), derived from an Iranian strain of *L. infantum,* compared to direct agglutination test (DAT) for the detection of VL in humans and domestic dogs as animal reservoir hosts of the disease. A combination of rK26 and rK39 antigens and also CA was evaluated using indirect ELISA on serum samples of 171 VL confirmed humans (n = 84) and domestic dogs (n = 87) as well as 176 healthy humans (n = 86) and domestic dogs (n = 90). Moreover, 36 serum samples of humans (n = 20) and canines (n = 16) with other potentially infectious diseases were collected and tested for finding cross- reactivity. The results of ELISA were compared to DAT, currently considered as gold standard for the serodiagnosis of VL. The sensitivity and specificity, positive predictive and negative predictive values were calculated compared to DAT. The positive sera had previously shown a positive DAT titer ≥ 1:800 for humans and ≥ 1:80 for dogs. Analysis was done by MedCalc and SPSS softwares. Using the combination of rK26 and rK39 in ELISA, a sensitivity of 95.2% and a specificity of 93.0% % were found in human sera at a 1:800 (cut-off) titer when DAT-confirmed cases were compared with healthy controls; a sensitivity of 98.9% and specificity of 96.7%% were found at a 1:80 (cut-off) titer compared with DAT. A good degree of agreement was found between the combined rK39 and rK26-ELISA with DAT in human (0.882) and dog serum samples (0.955) by kappa analysis (*p* < 0.05). The ELISA using the CA test showed 75% sensitivity in human and 93.1% in dog serum samples as well as 53.5% specificity in human and 83.3% in dog,s sera, respectively. The combination of rK26 and rK39 recombinant antigen prepared from Iranian strain of *Leishmania infantum* showed high accuracy for the serodiagnosis of VL in human and domestic dogs. Further extended field trial with a larger sample size is recommended.

## Introduction

Visceral leishmaniasis (VL), is a systemic parasitic disease caused by protozoa of the *Leishmania donovani* complex. According to global health statistics, VL remains one of the more serious parasitic diseases with outbreaks and mortality potential. An estimated 50,000 to 90,000 new cases of VL occur worldwide annually. More than 90% of new visceral leishmaniasis (VL) cases were reported from 10 countries including Brazil, China, Ethiopia, India, Iraq, Kenya, Nepal, Somalia, South Sudan and Sudan in 2020^1^.

Mediterranean visceral leishmaniasis (MVL) caused by *Leishmania infantum* has been reported in northwest, southwest, southern, and northeastern parts of Iran^2, 3^. In VL-endemic areas of Iran, 90% of 1698 serologically positive VL cases were children younger than 12 years during 2002 to 2012^3^.

Dogs are considered to be a reservoir for *L. infantum* and have a main role in zoonotic transmission of MVL^4^. However, asymptomatic humans can also be responsible for anthroponotic transmission^5^. Then, early diagnosis of infection in dogs and humans is necessary in order to reduce the number of VL cases in endemic areas^6^.

Although, parasitological methods have 100% specificity for the diagnosis of VL, their sensitivity is relatively low and these methods are invasive^7^. Serological tests, based on antibody detection, such as direct agglutination test (DAT), immunofluorescence assay (IFA), an enzyme-linked immunosorbent assay (ELISA) are available. DAT is an appropriate tool for the serodiagnosis of human VL with high sensitivity and specificity^7^. As DAT is a simple, accurate and efficient serological test, it was recommended for serodiagnosis of human VL (HVL) as well as canine VL (CVL) particularly in endemic areas^8^.

Kinesin-related conserved recombinant antigens (i.e. rK39, rK26, rK9, rK28, rKLO8, rKRP42) have been tested for improved sensitivity and specificity in the detection of antibodies against VL^9, 10^. Moreover, the immunochromatographic tests (ICT) based on these antigens are very useful, low-cost, rapid, and practical in the field. ELISA tests based on rK39 and rK26 recombinant antigens have shown acceptable sensitivity and specificity in the studies performed on human and dog sera in different areas^11, 12^. Our previous investigation demonstrated that the recombinant K39 antigen from an Iranian strain of *L. infantum* has high sensitivity and specificity in symptomatic HVL and CVL^13^. Therefore, we decided to determine the sensitivity and specificity of a combination of rK39 and rK26 Ag prepared from an Iranian strain of *L. infantum* (MCAN/IR/14/M14 with GenBank Accession number **KT201383**) to detect anti-*Leishmania infantum* antibodies in both symptomatic and asymptomatic human and domestic dogs.

## Results

### Assessment of *L. infantum* mixed rK39 and rK26 recombinant antigen compared to DAT on human and domestic dog serum samples using ELISA

Altogether, 84 DAT positive sera from VL- infected humans with anti-*Leishmania* antibodies at ≥ 1:800, 87 DAT positive sera from VL- infected dogs with anti-*Leishmania* antibodies at ≥ 1:80 and 176 sera from healthy controls (86 human and 90 dog serum samples) were tested by the combination of *L. infantum* rK39 and rK26 and CA, respectively. It is necessary to mention that 36 serum samples from patients with other potentially cross-reactive infectious diseases including 20 infected human sera and 16 infected dog sera were assayed in the control group (Tables 1 and 2). In this study, 25 of 52 symptomatic humans with a DAT titer ≥ 1:3200 were also checked by parasitology methods (microscopy and culture).

**Table 1:** Comparison between DAT and mixed rK39 and rK26 on human serum samples.

Human sera	Mixed rK26 and rK39 ELISA		Total
	Negative	Positive	
*DAT^−^	80 (93%)	6 (7%)	86
DAT^+^	4 (4.8%)	80 (95.2%)	84
Total	84 (49.4%)	86 (50.6%)	170

*DAT^−^ contain healthy individuals (n = 66) and other infectious diseases (n = 20) as control group.

**Table 2:** Comparison between DAT and mixed rK39 and rK26 antigen results on canine serum samples.

Dog sera	rK26 mixed rK39 Mix-ELISA		Total
	Negative	Positive	
*DAT^−^	87 (96.7%)	3 (3.3%)	90
DAT^+^	1(1.1%)	86(98.9%)	87
Total	88(49.7%)	89(50.3%)	177

*DAT^−^ contain healthy dogs (n = 74) and other infectious diseases (n = 16) as control group.

Data analysis indicated that the total sensitivity and specificity of the rK39 and rK26 antigens used as a mixture*,* were 95.2% and 93% in humans and 98.9% and 96.7% in dogs, respectively; these values were lower in CA-ELISA with 75% and 52.3% in humans, 93.1% and 83.3% in dogs, respectively (Table 3). The sensitivity of the mixed rK39 and rK26-ELISA for sera from symptomatic (≥ 1:3200) (n = 52) and asymptomatic (≤ 1:1600) (n = 32) humans was 100% and 87.5%, respectively, with a 93% specificity. CA-ELISA showed a sensitivity of 85.2% for symptomatic individuals, whereas it had a sensitivity of approximately 59.4% for asymptomatic individuals. The CA-ELISA had a specificity of 53.5% in humans with high DAT titers (≥ 1:3200), but only 52.3% in humans with lower DAT titers (≤ 1:1600) (Table 4).

**Table 3:** Sensitivity, specificity, positive predictive value, and negative predictive value of Crude Ag- ELISA and mixed rK39 and rK26 ELISA tests in comparison with DAT for serodiagnosis of *L. infantum* infection on human and dog’s sera.

Host	Tests	Sn. (%)	Sp. (%)	PPV (%)	NPV (%)
Human	rK39 and rK26 -ELISA	95.2	93	93	95.2
	Crude Ag- ELISA	75	53.5	61.2	68.7
Dog	rK39 and rK26 -ELISA	98.9	96.7	96.6	98.9
	Crude Ag- ELISA	93.3	83.3	84.6	92.6

*Sn* Sensitivity, *Sp* Specificity, *PPV* Positive predictive value, *NPV* Negative predictive value.

**Table 4:** Sensitivity, specificity, positive predictive value, and negative predictive value of Crude Ag and mixed rK39 and rK26—antigens compared to DAT for serodiagnosis of symptomatic and asymptomatic VL patients.

Tests	VL patients	No	Sn	Sp	PPV	NPV
rK39 and rK26 -ELISA	Asymptomatic	32	87.5%	93%	82.4%	95.2%
	Symptomatic	52	100%	93%	89.7%	100%
Crude Ag- ELISA	Asymptomatic	32	59.4%	52.3%	31.7%	77.6%
	Symptomatic	52	84.6	53.5	52.4	85.2

*Sn* Sensitivity, *Sp* Specificity, *PPV* Positive predictive value, *NPV* Negative predictive value.

McNemar's test revealed no significant differences between mixed rK39 and rK26—ELISA and DAT results in humans *P* = 0.745 and dogs *P* = 0. 625. For human samples, there was a significant difference when comparing the CA-ELISA to the DAT (*P* = 0.02), but not for dogs (*P* = 0.078).

The *k* index was used to assess the amount of agreement between mixed rK39 and rK26 and CA-ELISA and DAT using SPSS software. In asymptomatic humans, there was complete agreement between mixed rK39 and rK26 -ELISA and DAT (*k* = 0.790), but no concordance of CA-ELISA with DAT (*k* = 0.092); in symptomatic humans, the degree of agreement was very high using the mixed rK39 and rK26 ELISA (*k* = 0.909), but weak in CA-ELISA (*k* = 0.340).

The *k* coefficient for mixed rK39 and rK26 ELISA and CA-ELISA in humans was 0.882 and 0.284, respectively. In dogs, these agreement values revealed nearly perfect agreement for mixed rK39 and rK26, as well as a substantial result for CA with DAT (*k* = 0.995 for mixed rK39 and rK26—ELISA and *k* = 0.763 for CA-ELISA) (Table 5).

**Table 5:** Sensitivity, specificity, positive predictive value, and negative predictive value of Crude Ag and mixed rK39 and rK26—antigens compared to DAT for serodiagnosis of symptomatic and asymptomatic VL infected dogs.

Tests	VL infected dogs	No	Sn. (%)	Sp. (%)	PPV (%)	NPV (%)
rK39 and rK26 -ELISA	Asymptomatic	3	100%	96.7	50	100
	Symptomatic	84	98.9%	96.7	96.5	98.8
Crude Ag- ELISA	Asymptomatic	3	50%	85.7	16.6	96.7
	Symptomatic	84	96.5	85.7	95.6	96.7

*Sn* Sensitivity, *Sp* Specificity, *PPV* Positive predictive value, *NPV* Negative predictive value.

**Figure 1:**
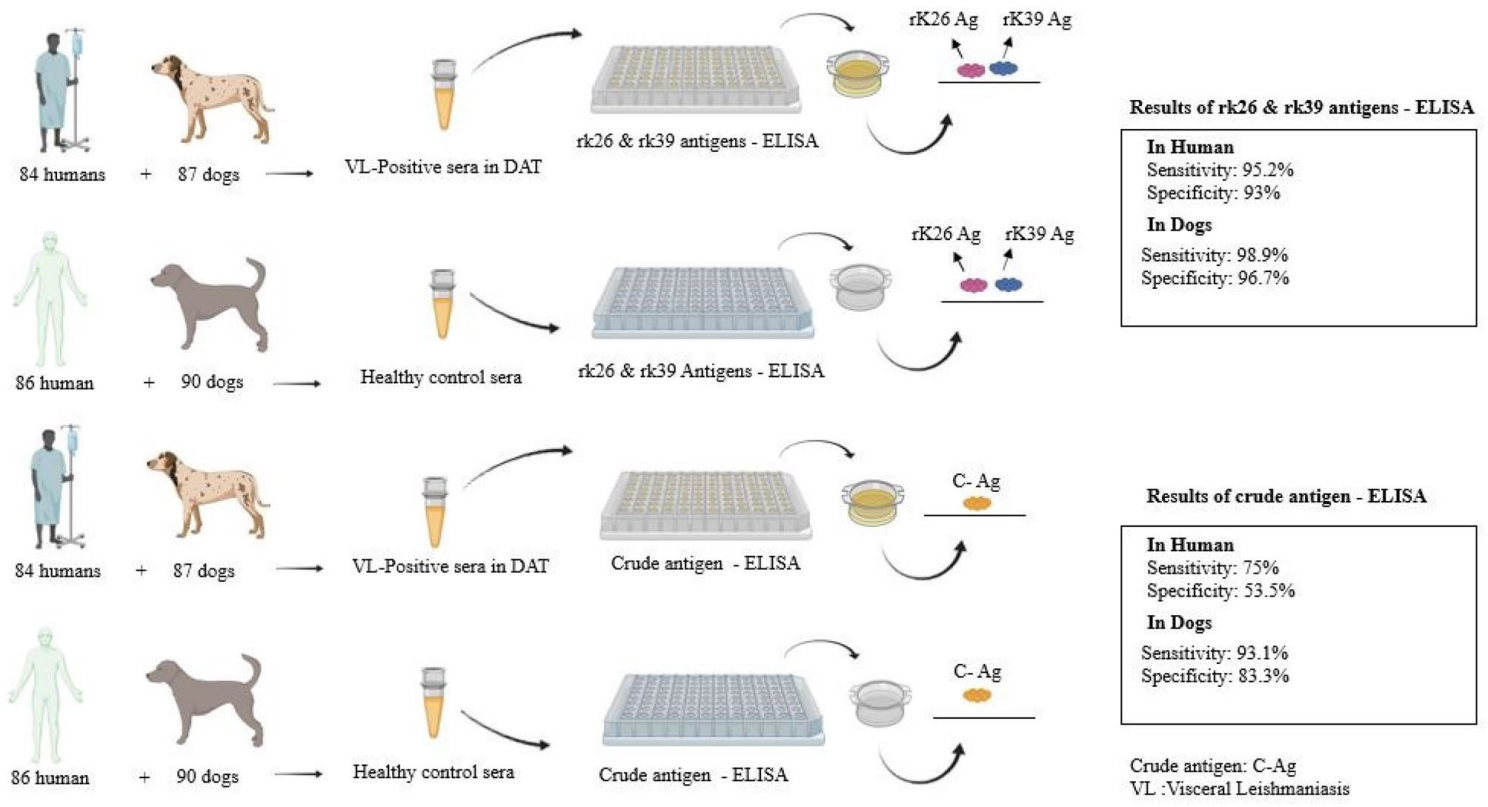
The results of the mixture of rK26 /rK39 and crude antigens from Iranian strain of *Leishmania infantum* to detect anti-*Leishmania* antibodies in human and reservoir hosts.

The Youden's indexes for mixed rK39 and rK26 -ELISA and CA-ELISA were 0.882 and 0.285 in human reservoirs, respectively, and 0.956 and 0.764 in dog reservoirs, indicating that mixed rK39 and rK26 -ELISA has a high accuracy in human and canine reservoirs, while CA-ELISA has an acceptable accuracy in dog (Fig. 1).

Cross-reactivity with the sera from individuals with other infectious diseases was not seen in the mixed rK39 and rK26 ELISA either in dogs or in humans. Six sera of healthy people were positive and reacted with the mixed antigens. Cross-reactivity of VL-negative human sera infected by cutaneous leishmaniasis (n = 4) and tuberculosis (n = 4) were seen for CA in this study.

## Discussion

The sensitivity and specificity of the diagnostic tests currently employed in Iran to assess CVL in asymptomatic and symptomatic VL in the field have been challenged, which has had a negative impact on the effectiveness of control measures^14^. Many endemic regions have heterogeneity of *Leishmania* parasites. In a particular population, tests based on homologous *Leishmania* antigens are required to detect cases that are difficult to diagnose with currently available tests^15^.

The current study shows that combining rK39 and rK26 from an Iranian strain of *L. infantum* in a single-well test has a high sensitivity and specificity for VL diagnosis in both symptomatic and asymptomatic reservoirs*.* This test proved to be superior to CA ELISA in terms of specificity, sensitivity and agreement with DAT.

This study builds on our previous results where we had already shown the recombinant rK39 antigen from an Iranian strain of *L. infantum* (MCAN/IR/14/M14 with GenBank Accession number **KT201383**) showed a high sensitivity and specificity at identifying human and dog cases with symptomatic VL^13^.

rK39 antigen is a 39-amino-acid repetitive immunodominant B-cell epitope of the 230 kDa kinesin related protein of *L. chagasi* and rK26 hydrophilic antigens has 11 copies of a 14-amino-acid repeat^16^. Recently, Farajnia et al. had described a high sensitivity (96.8%) and specificity (100%) when using rK26 produced from an Iranian strain of *L. infantum* in VL serodiagnosis^17^. However, when rK26 from *L. donovani* strain was examined in the Indian subcontinent, the sensitivity of the test dropped to only 38%, while in Brazil, only 56% of cases of VL were found to be positive^16, 18^.

Dogs with clinical signs of leishmaniasis are the main reservoir of *L. infantum* for humans.

In areas where zoonotic VL is endemic, the prevalence of *L. infantum* in dogs is often high while many of them are asymptomatic. Domestic dogs infected by VL play an important role in transmission of VL to humans^19^. The control of VL depends on early case detection in humans and adequate treatment. Diagnostic tests play a major role in the control of VL, as well as patient management, screening of infections, and epidemiological studies^20^. Since some evidence is available on the great sensitivity of the rK26 antigen in the diagnosis of VL at the early stage of infection, we decided to mix the rK39 antigen with rK26 derived the same Iranian strain of *L. infantum* to diagnose VL in human and dog^21, 22^.

We showed that rK39 and the rK26 carry immunodominant epitopes which can be useful for the serological diagnosis of canine and human visceral leishmaniasis.

The antibody reactivity and sensitivity of these two recombinant antigens were high when they were employed in parallel, suggesting that combining them in a single-well test could improve the assay's performance even more^23^.

The results of this study demonstrated that a mixture of rK39 and rK26 ELISA could efficiently detect antibodies in infected cases achieving a high sensitivity of 95.2% for human sera and 98.9% for dog sera. A study in Brazil reported a similar sensitivity (100%) using rK39 and rK26 antigens by RDT (TRALd) in symptomatic CVL diagnosis^24^.

In present study, mixed rK39 and rK26 ELISA has shown approximately 100% sensitivity in cases of parasitologically confirmed VL and high antibody-titer in dogs and humans similarly to what seen in the rk39 ELISA^13^. There was statistically significant correlation between the antibody titer and sensitivity^25–28^. There are a variety of factors affecting the levels of antibody production, including the load and species of infecting parasite, the form of the disease, the genetic background of the host and vector-derived products^29^. Sex and age are additional factors that might affect the antibody response, but this was beyond the scope of this study.

Moreover, a meta-analysis of published data revealed that the rK39 RDT has a lower sensitivity in dogs with clinical infection (87%) than in symptomatic humans (94%) infected with *L. donovani* or *L. infantum*^27^. Our data indicated mixed rK39 and rK26 increases the sensitivity of the test in symptomatic CVL (98.9%). It is possible that this is due to the increase of rK39 and rK26 immunodominant epitopes in this ELISA test and more ability of anti-*Leishmania* antibodies in reservoirs to bind them.

The results of rK39/rK26 ELISA showed a high sensitivity rate of 87.5% in asymptomatic HVL. Low sensitivity of rK39-ELISA (62.5%) has been reported in self–healing or sub-clinical human infection in our previous study^13^.Other studies on asymptomatic HVL described similar sensitivity 62.5% using rK39-ELISA^30, 31^. A significant 25% increase in diagnostic sensitivity in the multiple epitopes format (rK39/rK26) ELISA was seen for asymptomatic cases, indicating the effectiveness of adding the rK26 antigen to rK39 in a single well.

Although the level of antibody titer is directly related to the tissue parasitic load and positivity rate in serological test, the results of the current study indicated that mixture of rK39 and rK26 increase the sensitivity of diagnosis in subclinical forms. In present study, there were only 3 dogs without clinical signs and all of them had a positive result for mixed rK39 and rK26 ELISA.

The *k* indexes between the rK39/rK26 ELISA and DAT were in complete agreement for the asymptomatic group (*k* = 0.790) and in perfect accordance in the human symptomatic group (*k* = 0.909) and dog reservoir (*k* = 0.995).

Although, lower sensitivity was reported for rK39/ rK26 ELISA in asymptomatic human cases than in symptomatic human ones in this study, all negative samples in the asymptomatic group were related to the cut-off antibody titer (1:800) and all asymptomatic human cases with anti-body titer 1:1600 have been diagnosed as VL positive.

In the case of the canine reservoirs, only three cases were asymptomatic, so a separate analysis was not performed for the asymptomatic group, but the three dogs with antibody titers of 1:160 were positive.

In patients with clinical symptoms, CA-ELISA had a sensitivity of 93.1% for CVL and 84.6% for HVL, respectively, but lower positivity rates of almost 59.4% were detected in sera with lower levels of antibodies (≤ 1:1600). Porrozzi et al. also reported a low CA-ELISA sensitivity (30%) in this group^25^. CA-ELISA has a higher sensitivity in symptomatic canines when compared to the same situation in human.

Surprisingly, despite a significant increase in sensitivity (84.6%) for CA-ELISA in HVL with clinical signs as compared to another group, it fails to achieve a good agreement in the connection with DAT (*k* = 0.340). Conversely, this value is acceptable in dogs with a high antibody titer (*k* = 0.763).

This study shows a 93% specificity for mixed rK39 and rK26 Ag in human, while the test specificity in the previous study, which was used rK39 Ag alone in the ELISA diagnostic wells, was 86%^13^. This 7% rise in specificity could be the effect of adding rK26 Ag and the increase in specific epitopes for detection of antibodies against *L. infantum*. So, it indicates the possibility of less cross-reactivity with the serum of healthy individuals.

Our results indicate the lower specificity (93%) of ELISA using mixed rK39 and rK26 Ag in human in comparison with specificity reported from Brazil (98%)^24^.This value was determined 96.7% when mixed rK39 and rK26 -ELISA was used to detect antibody in the canines. Boario et al. stated a 99% specificity in both human and canine control groups using Chimeric (K9, K26, and K39 antigens) ELISA^32^. However, cross-reactivity was not seen for mixed rK39 and rK26 -ELISA in human and dog sera with other infectious disease sera applied in the current study, the less specificity of mixed rK39 and rK26 has been reported in comparison with other researches. It is likely due to higher prevalence of other infectious diseases with similar signs.

None of the sera from individuals with other infectious diseases produced a positive reaction in the mixed rK39 and rK26 ELISA, while cross-reactivity of VL-negative sera infected by cutaneous leishmaniasis and tuberculosis were seen for CA in this study. Therefore, in contrast to CA-ELISA, the mixed rK39 and rK26 -ELISA can discriminate true leishmanial infection with more specificity.

Furthermore, when compared to combined rK39 and rK26, CA has a lower specificity (53.5% in humans and 83.3% in dogs). Reactions with healthy blood donors' sera and cross-reactivity with other infections could be the main^33^.

Overall, the high validation of the mixed rK39 and rK26 antigens comparable to DAT suggests that combination rK39 and rK26 seems to be a good option for diagnosing symptomatic and asymptomatic VL infected humans and dogs. CA-ELISA has a poor agreement with the gold standard DAT in humans, but an outstanding agreement with DAT in dogs.

Future efforts to optimize the binding of mixed rK39 and rK26 antigens to nitrocellulose strips could result in a speedy and cost-effective dipstick test that would change VL diagnosis in Iran and other countries where *L. infantum* is the predominant causative agent. Furthermore, this test improves the sensitivity of the assays for diagnosing VL in the asymptomatic population in Iran.

## Methods

### Ethics approval and consent to participate

This study was approved by the Ethical Committee of Tehran University of Medical Sciences (IR.TUMS.SPH.REC.1397.203 and 92-03-162-24558) in accordance with the Helsinki Declaration and guidelines. Dog sera were collected in coordination with Iran Veterinary Organization after informed consent from the owners. The human sera were collected from volunteers following informed consent. Children were included in this study after consent from their legal guardians.

### Study population

The sample size was calculated based on the sensitivity and specificity of 70% obtained from studies done on rK39 dipstick test for diagnosis of VL in Iran^34^. The 95% confidence level with a margin of error of less than 10% was considered for this sample size.

Totally, 3 ml of 171 confirmed VL- infected sera with a positive DAT were collected between April 2016 and March 2018, using a non-probability convenience sampling method by Iranian Reference Laboratory for leishmaniasis in TUMS. The sera were kept frozen at − 20 °C after doing DAT until use in ELISA tests. All the serum samples including 84 humans and 87 dogs showed anti-*Leishmania* antibodies in DAT at ≥ 1:800 for humans and at ≥ 1:80 for dogs^35^.

Amastigotes were observed in bone marrow samples of 25 of 52 patients with active VL (with anti-*Leishmania* antibodies at ≥ 1:3200 titers); the remaining symptomatic patients (27 patients) showed clinical signs of VL including hepatosplenomegaly, anemia, and a prolonged irregular fever (n = 27). The number of asymptomatic patient sera with titer of anti-*Leishmania* antibodies less or equal to 1:1600 were 32.

Fifty-four serum samples were collected from dogs with pathognomonic clinical signs of VL including skin lesions, alopecia, diarrhea, and splenomegaly (without biopsy, n = 54). Amastigote forms were demonstrated in spleen and liver biopsy samples of 30 dogs with active VL (34.5%). All the collected sera from dogs with active VL showed anti-*Leishmania* antibody titers ≤ 1:320. Three dogs had parasitological positive biopsy samples with 1:160 anti-*Leishmania* antibody titers using DAT (n = 3).

In parallel, 3 ml serum sample were collected from 176 sera samples from healthy people in geographical areas non-endemic for VL and stored at − 20 °C. 86 humans at titers of anti-*Leishmania* antibodies ≥ 1:800 and 90 dog samples at titers of anti-*Leishmania* antibodies ≥ 1:80 determined by DAT. Moreover, 20 sera were collected from malaria, tuberculosis, cutaneous leishmaniasis, toxoplasmosis and hydatidosis patients and 16 canine sera positive for toxocariasis, toxoplasmosis, cutaneous leishmaniasis and babesiosis for determining of cross-reactivity.

### Preparation of mixed recombinant rK39 & rK26 antigens for ELISA

The inserted *L. infantum* k39 and k26 genes in pET 32a ( +) vectors were used for protein expression in BL21(DE3). The rK39 and rK26 proteins were purified from the soluble fraction by NI-IDA resin affinity chromatography. Western blotting revealed a single band of 58 kDa for rK39 and 50 kDa for rK26. Protein concentration has been estimated 47.8 µgr/ml and 19.8 µgr/ml for rK39 and rK26, respectively after purification and dialysis. All the protein expression and purification procedures were based on the protocols described by Hosseini et al.^13, 22^.

### Preparation of promastigote crude antigen for ELISA

The *L. infantum* strain (MCAN/IR/14/M14 with GenBank Accession number **KT201383**) used in this study was isolated from an infected dog with clinical signs from one of the endemic areas for VL in Iran. Spleen biopsy samples were aseptically cultured in RPMI-1640 (Sigma-Aldrich) and 10% inactivated fetal bovine serum (FBS) at 25 ± 1 °C. The late-logarithmic phase of *L. infantum* promastigotes in the first passage *(*MCAN/IR/14/M14 with GenBank Accession number **KT201383**) were sonicated after washing in cold PBS for 3 times. The supernatant containing crude Ag was stored at − 20 °C after centrifugation at 4500 rpm for 20 min at 4 °C. Protein concentration was estimated by the Lowry’s method^36^**.**

### ELISA assay

rK39 & rK26 antigens from an Iranian strain of *L. infantum* produced and purified as previously described were aliquoted and stored at − 70 °C until further use^13, 22^. The concentration of mixed rK39 & rK26 and CA was optimized by a checkerboard titration in standard ELISA methods^37^.

ELISA plates (Nunc™ MaxiSorp™) were coated overnight with mixed rK39 & rK26 Ag (1 + 1 µg/ml) and crude antigen (6 µg/ml) at 4 °C in carbonate/bicarbonate buffer at pH = 9.6. Then, the protocol was done according to the method Hosseini et al*.* explained.

The optical density (OD) was entered into MedCalc (Version 17.7.2). The ROC- curve was created for mixed rK39 & rK26 and CA in this software. The best cut-off points were selected to give the maximum sensitivity and specificity.

### Direct Agglutination Test (DAT)

Serial dilutions of sera (1:800 to 1:102,400 for human sera in physiological saline (NaCl 0.9%) and 1:80 to 1:20,480 for dog sera in physiological saline (NaCl 0.9%) to which 0.78% and 1.56% β – Mercaptoethanol for human and dog sera respectively, along with negative and positive control sera were added to each well of a V-shaped plate (NUNC, Germany). 50 µl of DAT antigen were added to each well and left overnight at room temperature. The agglutination was checked visually^17, 38^*.*

The cut-off point for human and dog sera were considered according to titers described by Hosseini farash et al. (1:800 for human and 1:80 for dog)^13^*.*

### Statistical analysis

The sensitivity and specificity, positive predictive and negative predictive values were calculated in comparison with DAT. To show the degree of agreement between expected result (mixed rK39 and rK26 Ag ELISA,) and actual result (DAT results), kappa (*k*) values (95% confidence intervals) were determined based on Landis and Koch criteria scales. The *k* is less than 0, no agreement, if 0–0.2, slight agreement, if 0.2–0.4, weak agreement, if 0.4–0.6, moderate agreement, if 0.6–0.8, substantial agreement and if 0.8–1.0, almost perfect agreement^39^. The correlation between mixed rK39 and rK26 Ag ELISA and CA-ELISA results with DAT was evaluated using McNamara's test and analyzed by MedCalc software (Version *17.7.2*). Youden's index was calculated to determine the diagnostic efficacy (accuracy), for mixed rK39 and rK26 Ag ELISA and CA-ELISA in comparison with the DAT. The datasets analyzed during the current study are available in the ImmPort Galaxy website and the history is currently accessible by visiting the following URL: https://galaxy.immport.org/u/hoseinifr/h/unnamed-history.
